# Early Eye Organoids Demonstrate Cadherin and Semaphorin Disruption in *PAX6*-Related Aniridia

**DOI:** 10.3390/ijms27198449

**Published:** 2026-09-22

**Authors:** Nicola Cronbach, Gabriela Justynska, Cécile Méjécase, Philippa Harding, Jonathan Eintracht, James Boot, Elsa Berg, Dulce Lima Cunha, Mariya Moosajee

**Affiliations:** 1 Institute of Ophthalmology, University College London, London EC1V 9EL, UK; nicola.cronbach.23@ucl.ac.uk (N.C.);; 2 Department of Molecular Developmental Biology, Radboud Institute for Molecular Life Sciences (RIMLS), 6525 GA Nijmegen, The Netherlands; 3 The Francis Crick Institute, London NW1 1AT, UK; 4 Department of Human Genetics, Radboud University Medical Centre, 6525 GA Nijmegen, The Netherlands; 5 Moorfields Eye Hospital NHS Foundation Trust, London EC1V 2PD, UK

**Keywords:** PAX6, aniridia, RNA-seq, hiPSC, organoids, cadherins, semaphorins

## Abstract

The paired box transcription factor PAX6 is a master regulator of eye development. Pathogenic *PAX6* variants predominantly cause aniridia, resulting in a wide range of ocular anomalies affecting both the anterior and posterior segments of the eye. However, a detailed examination of the transcriptomic alterations that occur with these variants during early eye development has not yet been undertaken. To investigate this, human-induced pluripotent stem cells (hiPSC)-derived 3D optic vesicle-like organoids were generated up to day 35 from an aniridia patient with a heterozygous nonsense PAX6^R261X^ variant. Bulk RNA sequencing analysis revealed dysregulation of genes in the cadherin and semaphorin receptor binding pathways in PAX6^R261X^ optic vesicles compared with healthy controls, including upregulation of *SEMA3A* and *CDH1* and downregulation of *CDH4*. These genes are known to be involved in iris and foveal development but have not previously been found to be dysregulated at the optic vesicle stage, suggesting they have a spatiotemporal impact on eye development. This study reveals a novel link between the cadherins, semaphorins and aniridia and suggests a novel pathway that may contribute to iris and foveal hypoplasia in patients with pathogenic *PAX6* variants. Expanding our knowledge of the complex interactions governing early eye development provides novel candidates for the development of potential targeted therapies and can help to guide translational research.

## 1. Introduction

The paired box transcription factor, PAX6, is known to play a key role in early eye development, and its dysregulation results in a wide range of ocular anomalies in both the posterior (retina and optic nerve) and anterior (cornea, lens, iris and ciliary body) segments of the eye [1].

PAX6 (OMIM #607108) acts throughout oculogenesis, initially as an eye field transcription factor in the optic vesicle, before stimulating optic cup patterning and the development of the lens placode [2,3,4,5,6]. It plays a central role in the development of the anterior segment, regulating neural crest cell migration to form the corneal stroma and endothelium, and subsequently the ciliary body and iris stroma [4]. PAX6 stimulates separation of the lens vesicle from the overlying developing cornea in conjunction with *CITED2* and E-, N- and P-cadherin [7,8,9]. Concurrent with the second wave of periocular mesenchymal migration into the anterior chamber to form the ciliary body and iris stroma, elongation of the peripheral optic cup tips to form the ciliary body and iris epithelium occurs [2,10]. The optic cup tips have the highest expression of *PAX6* and also express *CYP1B1* and *MEIS2* [5,11,12]. Notably, studies of anterior segment development have found that *PAX6* expression is high and continuous in cells of ectodermal origin (corneal, iris and ciliary body epithelia and lens) but cells originating from the periocular mesenchyme (corneal stroma and endothelium, iris and ciliary body stroma and trabecular meshwork) express *PAX6* transiently and at a much lower level [2,3,5,13].

Aniridia is a pan-ocular disease which may occur in isolation or in association with Wilms tumour as part of the WAGR syndrome (Wilms tumour, aniridia, genital anomalies and a range of developmental issues). Classical aniridia (OMIM #106210) includes foveal and iris hypoplasia with resultant nystagmus and reduced visual acuity, often associated with lenticular opacities, juvenile- or adult-onset glaucoma and aniridia-associated keratopathy [1,14]. Other ocular features may include optic nerve hypoplasia, strabismus, microphthalmia, anophthalmia and coloboma spectrum, vascular retinopathy and choroidal degeneration. The condition is also associated with an array of systemic features, including olfactory deficits, hearing impairment, obesity, and behavioural and sleep disorders. Dominant heterozygous *PAX6* mutations are identified in 90% of aniridia patients with a genetic diagnosis [1]. Approximately 70% of cases are due to the introduction of a premature termination codon in *PAX6* as a result of a nonsense or frameshift mutation [15]. Missense variants in *PAX6* have a highly variable phenotype and whilst some patients can present with isolated foveal hypoplasia, others have aniridia, corneal changes or rarely severe microphthalmia [14,16,17].

Techniques to culture ocular organoids from human-induced pluripotent stem cells (hiPSCs) as in vitro models of disease have broadened our ability to study human eye development and the effects of specific germline mutations on underlying molecular processes [18,19]. Specific culture conditions can guide hiPSC differentiation into distinct ocular structures, such as cornea, lens, retina and retinal pigment epithelium (RPE) [19,20,21,22,23]. However, at early stages of culture, progenitors of multiple different ocular tissues develop together, with co-cultures expressing markers for surface ectoderm, neural crest, lens, neural retina, RPE and ciliary margin, as shown by transcriptomic and immunochemistry analysis [21,22,24]. By day 20 of differentiation, early ocular organoids resemble the cellular composition and molecular expression profile of the week 4.5 human optic vesicle, with early lamination becoming apparent, and expression of retinal progenitor markers *PAX6*, *RAX* and *OTX2*, in addition to optic cup markers *JAG1* and *CITED2*, and ocular surface markers *TFAP2A*, *KRT15* and *TP63*/*PAX6* co-expression [18,20,24]. By day 35, the organoids resemble developmental week 6 early optic cups with layering of the neural retina and RPE progenitors expressing *PAX6*, *VSX2*, *MITF* and *SOX2*, in addition to lens markers *CRYAB*, *CRYBB1* and *MAF* [18,19,21,24]. These early ocular organoids can therefore be utilized to examine the molecular effects of pathogenic variants on the initial stages of eye development, providing insights into optic cup, retina and anterior segment formation.

We have previously generated hiPSC-derived early ocular organoids from an aniridia patient carrying a *PAX6* nonsense variant, c.781C > T p.(Arg261*) (PAX6^R261X^), where we showed that aniridia optic vesicles produced increased expression of neural retina and proliferation markers *VSX2* and *MKI67*, respectively [19]. In a subsequent study, we elucidated the transcriptomic differences between optic vesicles derived from a patient with microphthalmia caused by a *PAX6* missense variant, c.372C > A p.(Asn124Lys) (PAX6^N124K^) and compared this to PAX6^R261X^ and wild-type (WT) samples [17]. This revealed downregulation of *SOX2* and Notch signalling components in PAX6^N124K^ samples compared with both PAX6^R261X^ and WT samples, with differences in *PAX6* and *SOX2* co-binding and reduced Notch pathway-related transcription factor binding in PAX6^N124K^ optic vesicles. However, a detailed examination of the molecular changes in the aniridia PAX6^R261X^ model during early eye development has not yet been undertaken. In this study we utilized bulk RNA-seq to identify transcriptomic disruption in hiPSC-derived PAX6^R261X^ optic vesicles and found evidence of dysregulation of cadherin and semaphorin receptor binding pathways in aniridia.

## 2. Results

To elucidate the molecular pathways that are dysregulated in *PAX6* aniridia, bulk RNA-seq analysis of day 20 and day 35 PAX6^R261X^ aniridia optic vesicles compared with wild-type (WT) controls was undertaken. Results were filtered by base mean expression ≥ 10, absolute log_2_ fold change (LFC) ≥ 1 and adjusted *p*-value (padj) < 0.05. This gave a total of 1033 significantly upregulated and 1615 significantly downregulated genes in the PAX6^R261X^ optic vesicles at day 20 compared to WT controls, and 615 significantly upregulated and 569 significantly downregulated genes at day 35.

There is clustering of the WT and aniridia cell lines at day 0, with separation by both time point and cell line at day 20 and day 35 (Figure 1, Supplementary Figure S1). There is no separation by clone (Supplementary Figure S2). There are differences in the expression patterns of optic cup and retinal progenitor markers between the PAX6^R261X^ aniridia and WT organoids at both day 20 and day 35. At day 20, the optic cup markers *JAG1* and *CITED2* are significantly downregulated in the PAX6^R261X^ samples compared with WT, as is the retinal progenitor marker *SOX2*, and there is relative downregulation of the retinal progenitor markers *RAX*, *OTX2* and *PAX6* itself. At day 35, when layering of the primitive retina is becoming apparent, *CITED2* remains significantly downregulated in the PAX6^R261X^ optic vesicles; there is relative upregulation of the neural retinal marker *VSX2* and the RPE marker *MITF*, while *PAX6* and *SOX2* remain downregulated (Supplementary Figure S1).

Consistent with previous work by this group in PAX6^R261X^ optic vesicles [19], these results show no significant difference in expression of the nonsense-mediated decay (NMD) activator *UPF1* in the aniridia samples compared with WT, suggesting NMD escape for the *PAX6* c.781C > T p.(Arg261*) variant. However, *PAX6* haploinsufficiency limits protein production, and we have previously demonstrated a significant reduction in PAX6 protein to less than half of wild-type levels at day 35 in this PAX6^R261X^ aniridia organoid model [19]. As PAX6 is one of the key transcription factors in early eye development, this impacts downstream pathways, with enrichment and overrepresentation analysis of this data highlighting differential expression within pathways relating to transcription (GO:0003899, GO:0034062, GO:0097747) and translation (GO:0019843, GO:0043022, R-HSA-72613, R-HSA-72649, R-HSA-72766, R-HSA-72737).

### 2.1. Dysregulation of Retinal and Foveal Genes Is Identified at an Early Stage in Aniridia-Derived Optic Vesicles

Dysregulation of genes involved in the formation of the retina and fovea is seen in these early PAX6^R261X^ optic vesicles. At day 20, there is significant dysregulation of genes involved in retinal lamination (upregulation of *CALB1*; downregulation of *DSCAM*, *PTF1A*, *SDK1*, *SDK2*), RPE development (upregulation of *MITF*; downregulation of *IHH*, *MFSD2A*, *PAX2*), neural retina development (downregulation of *ATP2B2*, *FOS*, *PER1*, *SLC17A7*) and photoreceptor cell development and differentiation (upregulation of *DIO1*, *HCN1*; downregulation of *BHLHE23*, *CRB2*, *FSCN2*, *NOTCH1*, *NTRK2*, *OLFM3*, *TH*, *VEGFA*). At day 35, *SDK2* and *TH* remain significantly downregulated, and there is significant upregulation of *ATP8A2* from the retinal lamination gene set.

Specific to foveal development, there is significant upregulation of *ANXA2*, *DIO2*, *FRZB*, *HOTAIR1* and *LUM* at day 20, with significant downregulation of *CDKN1A* at this point. At day 35, none of these genes remain significantly dysregulated, but there is instead significant upregulation of *CD44* and *HKDC1*, and significant downregulation of *CRYAB*.

There is no significant dysregulation of genes involved in retinal vascular development seen at day 20. However, at day 35 there is significant upregulation of *ACVRL1* and *CYP1B1*, with significant downregulation of *ARHGEF15*.

### 2.2. Dysregulation of Anterior Segment Genes Is Identified at an Early Stage in Aniridia-Derived Optic Vesicles

A comparison of the WT day 0 organoid data to WT day 20 and day 35 data was done, focused on genes pertaining to human anterior segment development (Figure 2, Supplementary Table S1). This demonstrates the differential expression of these genes at both day 20 and day 35 in WT optic vesicles and confirms that the dysregulation seen in these genes in the aniridia samples can confidently be used to infer impact on anterior segment development at this early developmental stage.

At day 20, analysis of PAX6^R261X^ optic vesicles showed dysregulation of genes involved in the formation of anterior segment tissues, as derived from gene ontology listings and published literature (Figure 3). Differential expression of genes involved in ocular surface/corneal development includes significant upregulation of *ANXA2*, *EPHA1*, *KRT15*, *PRICKLE1* and *WNT6*. Additionally, there was non-significant upregulation of *BMP4* and *TP63* and downregulation of *TFAP2A* and *PAX6* expression at this time point. Expression of the early corneal stromal markers *LUM*, *DCN* and *COL5A2* was significantly upregulated, whilst *COL6A1* expression was significantly downregulated. Differential expression of genes involved in iris development includes significant upregulation of *MEIS2* and *WNT2* and significant downregulation of *OTX1* and *LMX1B*. Differential expression of genes involved in ciliary body development includes significant upregulation of *DCN* and significant downregulation of *JAG1* and *SLC6A1*. Differential expression of genes involved in lens development includes significant upregulation of *BFSP1*, *PLAAT1*, *PLAAT3*, *TDRD7* and *WNT2* and significant downregulation of *CITED2*, *EPHA2*, *HSF4*, *SOX1*, *VIM*, *WNT7A* and *WNT7B*.

At day 35, the PAX6^R261X^ optic vesicles demonstrated persistent significant upregulation of *KRT15* and significant downregulation of *CITED2*, *COL6A1*, *LMX1B*, *SOX1* and *WNT7B*, with additional significant upregulation of the iris-related genes *CYP1B1* and *WNT16* and significant downregulation of the lens marker *CRYAB* (Figure 4). Although non-significant, there was relative upregulation of the lens marker *CRYBB1* and downregulation of *MAF* in the day 35 aniridia organoids compared with WT.

### 2.3. Cadherin and Semaphorin Pathways Are Dysregulated in PAX6^R261X^ Aniridia Optic Vesicles

Gene ontology overrepresentation analysis revealed that cadherin binding (GO:0045296) and semaphorin receptor binding (GO:0030215) were significantly overrepresented in the PAX6^R261X^ optic vesicles compared with the WT samples (Figure 5).

Of the 337 genes comprising the cadherin binding pathway, 35 genes were differentially expressed in the PAX6^R261X^ optic vesicles. These include significant upregulation at day 20 of *CDH1* (E-cadherin), *CDH3* (P-cadherin), *ANXA1*, *ANXA2*, *CDH22*, *CAPG*, *CGN*, *COBLL1*, *DOCK9*, *EIF3E*, *EZR*, *GPRC5A*, *HSPA8*, *KRT18*, *LAD1*, *LIMA1*, *PAK6*, *PDLIM1*, *PKP2*, *PKP3* and *ZC3HAV1*; and significant downregulation of *CDH4* (R-cadherin), *CDH8*, *CDH23*, *ATXN2L*, *CDK5R1*, *CNGA3*, *CTNND2*, *DCHS1*, *EPHA2* and *PLEC*. At day 35, the cadherin binding pathway genes that remain significantly upregulated are *CDH1*, *CAPG*, *GPRC5A*, *KRT18* and *PKP3*, and *CDH4* remains significantly downregulated. There was additional significant upregulation of the cadherin binding pathway genes *ARHGAP18*, *PKP1* and *SFN* noted at day 35 (Figure 6, Supplementary Table S2).

Of the 24 genes comprising the semaphorin receptor binding pathway, 11 genes were differentially expressed in the PAX6^R261X^ optic vesicles. These include significant upregulation at day 20 of *SEMA3A*, *SEMA3C*, *SEMA3D* and *SEMA3E*, and significant downregulation of *SEMA3F*, *SEMA3G*, *SEMA4A*, *SEMA4D*, *SEMA5B*, *SEMA6C* and *RIT2*. At day 35, *SEMA3A*, *SEMA3C* and *SEMA3E* remain significantly upregulated and *SEMA3G* remains significantly downregulated (Figure 7 and Figure 8).

In addition to the cadherins and semaphorins, other cellular adhesion markers were dysregulated in the aniridia optic vesicles compared with WT. Expression of the transmembrane cellular adhesion glycoprotein *L1CAM* was significantly downregulated at both day 20 and day 35 (Figure 8). Significant upregulation of the claudins *CLDN4*, *CLDN6*, *CLDN10* and *CLDN11* was found at day 20, with *CLDN4* and *CLDN6* remaining significantly upregulated at day 35. Similarly, the integrin *ITGA1* was significantly upregulated at day 20 and day 35 in the aniridia samples, while *ITGA2B* and *ITGA5* were significantly downregulated at both time points. At day 20, *OCLN* (occludin) was significantly upregulated and *NCAM1* was significantly downregulated; they remained up- and downregulated, respectively, at day 35, but the dysregulation was no longer significantly different from the WT samples (Supplementary Figure S1).

### 2.4. CDH1 and EPHA2 Proteins Are Dysregulated in PAX6^R261X^ Early Ocular Organoids

Although there was no significant difference in fold change on qPCR at day 35, immunofluorescence staining for CDH1 confirmed increased expression in the PAX6^R261X^ early ocular organoids in comparison to the wild-type organoids, with co-localisation with K18 but not with PAX6 (Figure 9, Supplementary Figure S3). Immunofluorescence staining for EPHA2 at day 35 is reduced in the PAX6^R261X^ early ocular organoids in comparison to the wild-type organoids, in keeping with bulk RNA-seq and RT-qPCR results (Supplementary Figure S4).

## 3. Discussion

This is the first study to report the transcriptomic changes occurring in patient-derived PAX6 haploinsufficient optic vesicles, contributing to our understanding of the molecular mechanisms underlying the phenotypic changes seen in aniridia. In addition to the disruption to retinal, foveal and anterior segment pathways, which were anticipated given the heterozygous PAX6 p.(Arg261*) nonsense mutation and clinical phenotype, we identified dysregulation of the cadherin and semaphorin binding pathways, neither of which has been previously linked directly to aniridia.

Molecular signals originating in the optic vesicle influence surface ectoderm and lens development from the third week of gestation, with *BMP4* and *LHX2* acting to induce development of the lens placode [25]. Retinoic acid and bone morphogenic protein released from the lens placode and surrounding extraocular mesenchyme stimulate invagination of the optic vesicle, following which *TGFß* from the extraocular mesenchyme and *FGF* from the surface ectoderm act to stimulate differentiation of the presumptive retinal pigment epithelium (RPE) and neural retina, respectively [25]. This study was undertaken on optic vesicles derived from hiPSCs. There is currently no hiPSC-derived organoid model that is able to reliably co-culture the developing neural retina with RPE, surface ectoderm and lens progenitors to form a true model of human oculogenesis. Furthermore, the model used herein has examined optic vesicles at day 20 and day 35 of development, which is far in advance of when the anterior segment truly begins developing [26], and the genes expressed at different time points throughout oculogenesis change in a precise and coordinated manner to ensure this progresses correctly. However, the use of these optic vesicles and the transcriptomic dysregulation seen with patient-derived mutations shed light on their potential impact on gene expression and development at later stages.

Many of the genes that regulate oculogenesis, including *PAX6*, are expressed at different time points throughout this process, with the precise combinations and balance of gene expression influencing different aspects of eye development. Dysregulation of the expression of one gene in the optic vesicle at day 20 or day 35 is likely to impact a different aspect of development than the same dysregulation at a later stage. For example, *CYP1B1*, *JAG1*, *LMX1B* and *MEIS2* are all involved in both anterior and posterior segment development and are all dysregulated in the PAX6^R261X^ optic vesicles.

*CYP1B1* is expressed in the neural retina, optic cup tips, iris and trabecular meshwork, as well as playing a role in retinal vasculature development [12,27]. *CYP1B1* mutations are known to cause primary congenital and juvenile glaucoma [28], Peters anomaly [29], aniridia [30] and other anterior segment anomalies [31,32]. *JAG1* contributes to optic cup patterning and is primarily associated with Alagille syndrome but mutations have also been identified in patients with Axenfeld–Rieger syndrome [33,34,35], of which developmental iris anomalies are a key feature. *LMX1B* mutations are associated with juvenile-onset glaucoma as part of Nail–Patella syndrome, but studies in zebrafish have shown that it is expressed in the optic fissure as well as in the periocular mesenchyme and presumptive anterior segment [36,37]. *MEIS2* is expressed by retinal progenitors at the optic cup stage in mice, then later in the ciliary margin zone and by retinal ganglion and amacrine cells [38,39]. Murine studies of *Meis1/2* double mutants reported that these mice have hypocellularity of the retina with coloboma, incomplete lenticular development, iris and ciliary body anomalies, and reduced expression of *Pax6* [38,40].

PAX6 interacts directly with *CYP1B1* [41] and *MEIS2* [3,42], and *JAG1* [43] and *LMX1B* [42] are predicted to have PAX6 binding sites. *CHI3L1* [44], *EDN3* [45] and the non-canonical *WNT* signalling molecules [46] have all been shown to be downstream of *PAX6*, although whether this is direct or indirect regulation is unclear, while *POSTN* is known to be mediated by *CYP1B1* [47]. This suggests that the phenotypic features seen in aniridia, including foveal and iris hypoplasia, early-onset glaucoma and ocular coloboma, are likely to be mediated in part through changes in *PAX6* modulation of the expression of these genes at the various stages of oculogenesis. This evidence of their interaction contributes to our knowledge of the complex molecular pathways governing different stages of anterior and posterior segment morphogenesis and differentiation, but further work in tissue-specific models is required to fully define these processes.

The cadherins are a family of Ca^2+^-dependent transmembrane glycoproteins which regulate cell–cell adhesion in numerous tissues and are essential for normal retinal lamination, neural crest cell migration and iris development [8,48,49]. Normal *CDH4* expression is necessary for proper retinal lamination, axonal pathfinding and synaptic formation among specific retinal ganglion cell (RGC) populations [50,51]; significant downregulation of *CHD4* was found in the PAX6^R261X^ optic vesicles at both time points. *CDH4* is co-expressed with *PAX6* in the ventral thalamus and the axons of the tract of the postoptic commissure during embryogenesis, and studies of *PAX6*^−/−^ mice have shown that loss of *PAX6* results in loss of R-cadherin expression in these regions [6,48]. Although foveal pit development does not begin until gestational week 25, the location of the future fovea is determined much earlier during oculogenesis by the development of the rod-free zone at the optic vesicle stage and subsequent development of the foveal avascular zone [52]. Patients with *PAX6* aniridia commonly present with foveal hypoplasia, and it has been suggested that the influence of *PAX6* on optic cup patterning, RPE differentiation and RGC development may all contribute to this [3,53], with the latter in particular resulting in a downstream reduction in pigment epithelium-derived factor and lack of formation of the foveal avascular zone (FAZ) [54]. *CDH4* downregulation resulting from *PAX6* haploinsufficiency is therefore a potential mechanism contributing to impairment of forthcoming foveal maturation and foveal hypoplasia, although further studies at later stages of ocular development are required to investigate this fully.

The precise expression patterns of E- (*CDH1*), N- (*CDH2*) and P- (*CDH3*) cadherin in the developing lens contribute to controlling the separation of the lens vesicle from the overlying corneal surface ectoderm, and repression of *CDH1* during chick neural crest development is required for induction of epithelial–mesenchymal transition (EMT), a process necessary for normal migration of iris cells to which *L1CAM* also contributes [8,55,56,57]. *CDH1* expression has been shown to negatively correlate with *PAX6* expression in non-small cell lung cancer cells [58], and knockout of *CDH1* from the lens vesicle in mice resulted in severe iris hyperplasia, microphthalmia and abnormal lens fibre cell differentiation and elongation [8]. Whether PAX6 is acting directly to modulate *CDH1* expression, or whether this is an indirect effect, is yet to be determined. *L1CAM*, which is directly regulated by PAX6 binding to its homeobox and paired domain-containing region [56], is associated with impairment of *CDH1*-related cellular adhesion and promotes cellular motility in breast carcinoma cells [57]. Here, we demonstrate significant upregulation of *CDH1* and significant downregulation of *L1CAM* in patient-derived *PAX6* aniridia optic vesicles. These results imply a conserved role for *PAX6* as an upstream negative regulator of *CDH1* and a positive regulator of *L1CAM*. Given this, we hypothesize that this pathway describes a potential mechanism by which *PAX6* haploinsufficiency may repress iris development resulting in iris hypoplasia and aniridia, although anterior segment studies at later stages of oculogenesis are required to confirm this.

The receptor tyrosine kinase EPHA2 is a member of the cadherin-binding pathway alongside *CDH1* and *L1CAM*, and contributes to global ocular growth and lenticular development during embryogenesis [59,60]. Pathogenic *EPHA2* variants are associated with autosomal dominant congenital cataracts and microphthalmia [59,61]; both phenotypes have been associated with *PAX6* aniridia, with childhood-onset cataracts reported in 63% of patients [62]. We demonstrate significant downregulation of *EPHA2* in our PAX6^R261X^ optic vesicles. Dysregulation of *EPHA2* resulting from *PAX6* haploinsufficiency could therefore be a potential mechanism influencing axial length in aniridia; coupled with dysregulation of other genes involved in lens morphogenesis (*CITED2*, *SOX1*), lens development (*WNT2*) and lens fibre cell development (*WNT7B*), this pathway may also contribute to cataract formation in these patients, although demonstration of these findings in lenticular tissue at an appropriate developmental stage is required for confirmation of this.

The semaphorin family of proteins shares an extracellular cysteine-rich sema domain and has multiple roles in embryogenesis, including retinal lamination, anterior segment formation and regulation of retinal and corneal angiogenesis [63,64,65]. *SEMA3A* is expressed by the neural retina, retinal angioblasts, lens epithelium, and iris and ciliary body mesenchyme, and is necessary for normal development of the retinal vasculature, cornea and anterior chamber [64,65,66,67]. *Sema3A* inhibition in chicks resulted in premature migration of neural crest cells into the anterior segment, lack of formation of the corneal endothelial monolayer and disordered corneal innervation and vascularisation [65,66,67]. Studies of *Pax6^Sey^*^/*+*^ mice have reported similar findings, with increased migration of neural crest cells into the anterior chamber during development with abnormal thickening of the corneal stroma and endothelium in adult mice [68], a feature that has also been reported in aniridia patients [69].

Along with *Sema3c* in mice, *sema3aa* in zebrafish has been shown to be regulated by *PAX6* [70,71]. The dysregulation of *SEMA3A* and *SEMA3C* in our PAX6^R261X^ optic vesicles indicates that *PAX6* acts upstream of these semaphorins during human embryogenesis, either directly or indirectly. Taken together with the findings from animal studies, this implicates *SEMA3A* dysregulation in the development of anterior segment anomalies in aniridia. Turning to the retina, *SEMA3A* is expressed by retinal angioblasts and is thought to contribute to the development of the retinal vasculature and FAZ [64], whilst *SEMA4A* mutations have been identified as a cause of retinitis pigmentosa and cone-rod dystrophy [72]. Dysregulation of *SEMA3A* and *CDH4* and their combined effects on the formation of the FAZ, particularly when coupled with *SEMA4A* disruption, describe a potential underlying mechanism for aniridia-related foveal hypoplasia which warrants further investigation.

The claudins and occludin are known to be key components in the formation and maintenance of tight junctions in both the blood–retinal barrier (BRB) and the cornea [73,74,75]. Our results show upregulation of *CLDN2*, *CLDN4*, *CLDN6*, *CLDN10* and *CLDN11* in the PAX6^R261X^ optic vesicles, all of which are present in the retina of wild-type mice with the exception of *CLDN6* [75]. *CLDN2* is known to be a component of the inner BRB, whilst *CLDN4*, *CLDN10* and *CLDN11* contribute to RPE tight junction integrity [75]. *CLDN2* and *CLDN4* have also been identified as components of corneal epithelial tight junctions in rabbits [76]. Aniridia-associated keratopathy (AAK) is known to be related to limbal stem cell deficiency and usually manifests clinically in adolescence and young adulthood in the mature cornea [77]. Our results from these PAX6^R261X^ optic vesicles, indicating that *PAX6* haploinsufficiency causes dysregulation of these claudins, raise the possibility that a similar effect in the cornea may contribute to the mechanism underlying AAK, but this cannot be proven with the model used herein. Studies in mature corneal models are required to further investigate this potential pathogenic mechanism.

Whilst this study has identified novel biological pathways contributing to the pathogenesis of aniridia, the limitations of this approach should be considered. As discussed above, this study utilized optic vesicles cultured from patient-derived hiPSCs, and we anticipate that the transcriptomic dysregulation resulting from *PAX6* haploinsufficiency reported herein may reflect similar dysregulation at other stages of ocular development. Although the use of this model for investigating retinal development is becoming widespread, it is not currently possible to co-culture neural retina with RPE, surface ectoderm/cornea and lenticular progenitors to form a true model of human oculogenesis, enabling us to study the myriad complex interactions that occur between these tissues.

This study utilized bulk RNA-seq of optic vesicles to assess the transcriptome-wide changes in gene expression in a *PAX6* nonsense mutation compared with wild type. Whilst single-cell RNA-seq provides resolution of variations in gene expression between individual cellular populations, bulk RNA-seq is more sensitive to subtle changes across the transcriptome and was therefore considered more appropriate for this study. Future work should utilize single-cell RNA-seq to help elucidate the influence of the dysregulation of the pathways identified herein on the developing cornea, iris, ciliary body and lens individually, both at this early developmental stage and in more mature models. Consideration should also be given to the design of studies to assess the impact of the optic cup tip-optic nerve *PAX6* expression gradient on the transcriptome in the developing neural retina and RPE.

In this study, one patient-derived aniridia line and therefore a single *PAX6* nonsense mutation was modelled, with two patient-derived wild-type lines for comparison. There is therefore the potential that line-specific variability may have influenced these findings. However, dysregulation of key genes reported in this paper was also identified in bulk RNA-seq of day 35 PAX6^N124K^ optic vesicles, including significant upregulation of *CDH1* and *SEMA3A* and significant downregulation of *CDH4* in PAX6^N124K^ organoids compared with wild type [17]. Whilst this supports *PAX6* haploinsufficiency as the cause of these changes, replication of the findings in isogenic cell lines is paramount to confirm this.

## 4. Materials and Methods

This study received local (Moorfields Eye Hospital, London, UK) and national (Northwest London, UK) research ethics approval and adhered to the tenets of the Declaration of Helsinki. Patients gave written informed consent for participation through the Genetic Study of Inherited Eye Disease (REC ref 12/LO/0141).

### 4.1. HiPSC Culture and Organoid Differentiation

Human-induced pluripotent stem cells (hiPSCs) were harvested from skin biopsies from the affected patient and healthy wild-type (WT) controls and were reprogrammed into hiPSCs using episomal vectors as described by Méjécase et al. [78] and Lima Cunha et al. [19]. The affected patient had a heterozygous nonsense variant in *PAX6*, c.781C > T p.(Arg261*), confirmed by Sanger sequencing, which is located in exon 10, within the homeobox domain [79]. They presented with a classic aniridia phenotype including iris and foveal hypoplasia, nystagmus and cataracts.

HiPSCs were cultured on Matrigel-coated plates (Corning Inc., Corning, NY, USA) in mTeSR Plus (STEMCELL Technologies, Vancouver, BC, Canada) and were passaged using ReLeSR (STEMCELL Technologies) when they reached 70% confluency.

Early ocular organoids were generated according to the protocol described by Eintracht et al. [18]. Briefly, on day 0, 0.5 mL of anti-adherence solution was added to each well of an AggreWell^TM^ 400 microwell plate (STEMCELL Technologies) before centrifuging at 1300 RCF for 5 min, washing with 2 mL DMEM and replacing with 1 mL mTeSR Plus supplemented with 10 μm Y27632 (STEMCELL Technologies). Cells were washed with PBS and incubated with Accumax (Sigma-Aldrich, St. Louis, MO, USA) for 5–8 min at room temperature to induce dissociation into single cells. 3.6 × 10^6^ cells were centrifuged and resuspended in 1 mL mTeSR/Y27632, then 3000 cells/microwell were added and centrifuged at 100 RCF for 3 min. On day 1, 1 mL of media was changed. On day 2, embryoid bodies were transferred to uncoated p60 dishes in Differentiation Media (DMEM/F12 + L-glutamine (Gibco, Thermo Fisher Scientific, Waltham, MA, USA), 2% B27 (Gibco), 1% NEAA (Gibco), 5 ng/mL IGF-1 (Sigma-Aldrich), 1% Penicillin/Streptomycin (Gibco) with 20% KOSR (Gibco) supplemented with 10 μm Y27632. Differentiation Media was changed on alternate days, with 15% KOSR from day 7, 10% KOSR from day 11 and supplementation with 10% FBS (Gibco), 0.1 mm Taurine (Sigma-Aldrich), 40 ng/mL Triiodothyronine (Sigma-Aldrich) and 0.5 μm Retinoic acid (Sigma-Aldrich) from day 18. To control for technical batch effects, two aniridia clones were cultured in parallel with two WT controls, with the same standardized methods and reagents used at each stage of the study.

### 4.2. RNA-Seq Library Preparation and Analysis

WT and PAX6^R261X^ samples taken at days 0, 20 and 35 were used for bulk RNA-seq analysis, with at least three biological replicates from each genotype at each time point included in the final analysis. RNA-seq library preparation was done by Novogene (Cambridge, UK) using the TruSeq Stranded Total RNA with Ribo Zero Plus rRNA depletion (Illumina cat 20020599) [17]. Sequencing was done using the HiSeq 2500 system with v4 chemistry (Illumina, San Diego, CA, USA) to an average depth of 85 million paired-end 150 bp reads with an average of 53% duplication.

Bulk RNA-seq analysis was done according to the nf-core/RNA-seq pipeline v3.10.1 [80]. FASTQC v0.11.9 [81] and TrimGalore v0.6.7 [82] were used for quality control and adapter trimming. One PAX6^R261X^ sample was removed from each of days 0 and 20 due to low RNA quantity. High-quality reads were aligned to the human genome GRCh38 [83] using STAR v2.7.10a [84] and quantified using RSEM v1.3.1 [85]. One PAX6^R261X^ sample was removed from day 0 due to low read count and high duplication rate. DESeq2 v1.42.1 [86] was used to find differential genes using the negative binomial distribution to model counts, with IHW v1.30.0 [87] for multiple-testing correction and ashr v2.2-63 [88] for effect-size shrinkage to ensure reported fold changes are more robust. Clone was included as a covariate in the analysis to confirm that batch effects did not influence the results.

Gene ontology (GO) [89] and Reactome [90] were used for gene set enrichment and over-representation analysis. Curated gene sets for anterior segment development were derived from relevant GO pathways pertaining to cornea, lens, iris, ciliary body and trabecular meshwork development (listed in Supplementary Table S1) and published literature [2,7,10,91,92].

### 4.3. RT-qPCR

Day 35 organoids were collected and RNA was extracted according to the Quick-RNA™ Microprep Kit (Zymo Research, Irvine, CA, USA). A total of 500 ng RNA was used for cDNA synthesis using the iScript cDNA synthesis kit (BioRad, Hercules, CA, USA). Used primer sequences are listed in Table 1. Quantitative PCR (qPCR) was carried out with iQ SYBR Green Supermix (BioRad) following the manufacturer’s instructions on a BioRad CFX96 system following standard cycling conditions.

Data were analyzed using the ΔΔCT method, normalized to the average expression of the housekeepers *GAPDH* and *ACTB*. Fold change values were calculated by normalizing to control (WT) samples and results were visualized using GraphPad Prism 10.6.0 (GraphPad Software, San Diego, CA, USA). Statistical significance of RT-qPCR data was determined using an unpaired Student’s *t*-test in GraphPad Prism (GraphPad Software). A *p*-value of <0.05 was considered statistically significant.

### 4.4. Immunofluorescence Stainings

Day 35 optic cups were collected using an EVOS XL imaging system (Thermo Fisher Scientific, Waltham, MA, USA), washed with DPBS (Gibco) and fixed with 4% PFA at 4C for 30 min. Samples were processed, sectioned and stained as previously described [17,18,19]. Slides were imaged with an Axio Imager M2 microscope (ZEISS Research, Oberkochen, Germany) and images were analyzed with Zeiss ZEN 3.12 software (ZEISS Research). Details of all antibodies used are shown in Table 2.

## 5. Conclusions

This is the first study to report a direct link between the cadherins, semaphorins and aniridia. This transcriptomic analysis of PAX6^R261X^ optic vesicles, derived from hiPSCs from a patient with aniridia, has demonstrated differential expression of genes in the cadherin binding and semaphorin receptor binding pathways, as well as in genes already known to affect anterior segment development. The dysregulation of *SEMA3A* and *CDH4* suggests a novel pathway that may contribute to foveal hypoplasia in patients with aniridia, whilst upregulation of *CDH1* and downregulation of *L1CAM* are particularly notable in this context. We demonstrate a potential mechanism by which *PAX6* haploinsufficiency may repress iris development resulting in iris hypoplasia and aniridia. Given the extensive overlap between causative genes and phenotypic features manifested in aniridia and other anterior segment dysgeneses [26], we hypothesize that these pathways may be dysregulated in other conditions causing anterior segment developmental anomalies.

Evidence of dysregulation of genes in these pathways and their interaction with *PAX6* expands our knowledge of the complex interactions between the many factors controlling early eye development, and future work should investigate the impact of these individual factors on phenotypic changes, both in aniridia and in other anterior segment dysgeneses. The findings reported herein should be strengthened by replication in isogenic controls, with single-cell RNA-seq to provide resolution of differential gene expression within individual cell populations. Furthermore, expanding the known downstream molecular targets of *PAX6* provides more candidates for future development of targeted therapies for related ocular diseases.

## Figures and Tables

**Figure 1:**
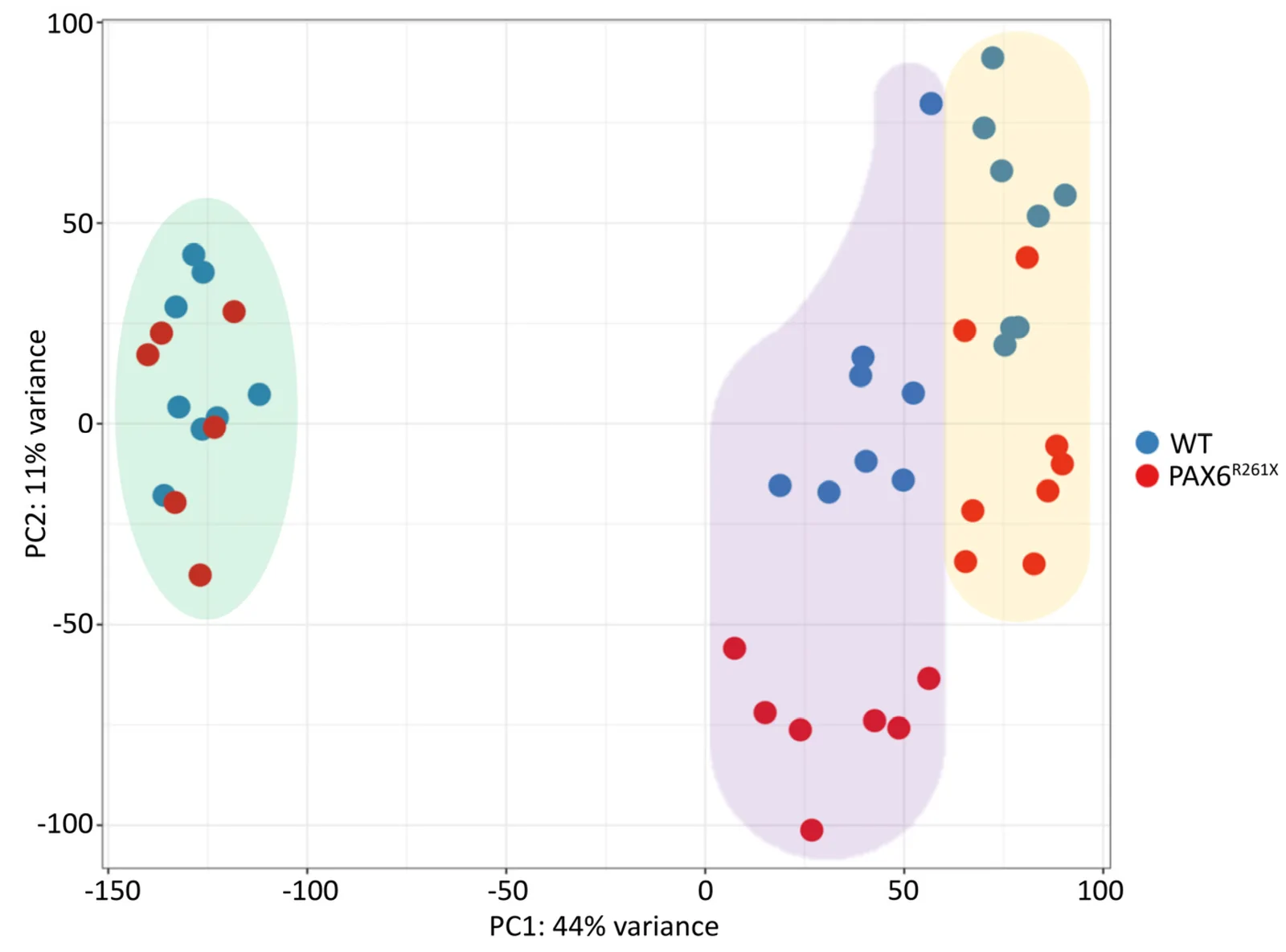
PCA plot by cell line and time point. Green area, day 0; purple area, day 20; yellow area, day 35.

**Figure 2:**
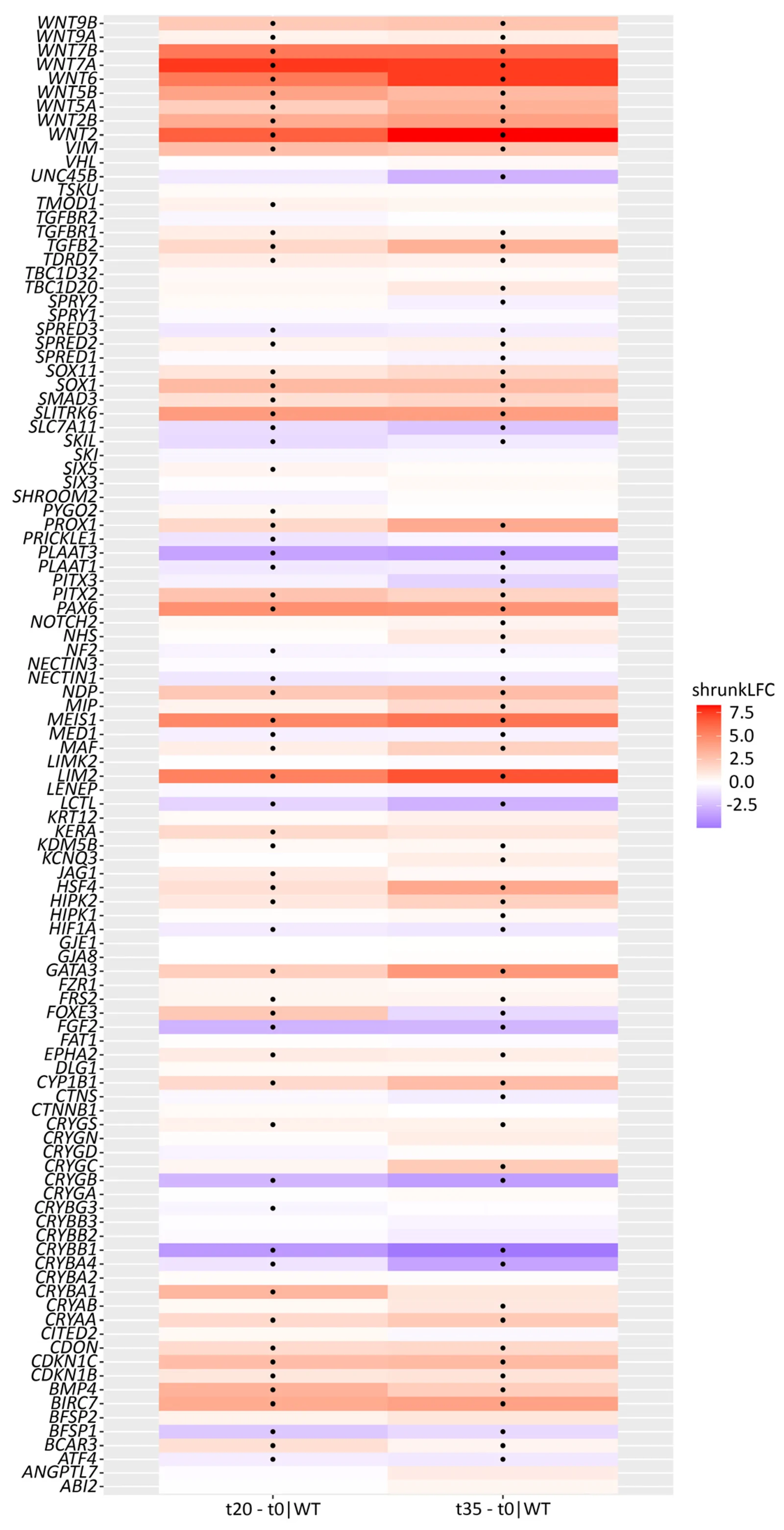
Heatmap showing differential expression of genes involved in anterior segment development in WT samples, with a black dot in the tile signifying significant differential expression of that gene in WT day 20 or day 35 compared with WT day 0 optic vesicles.

**Figure 3:**
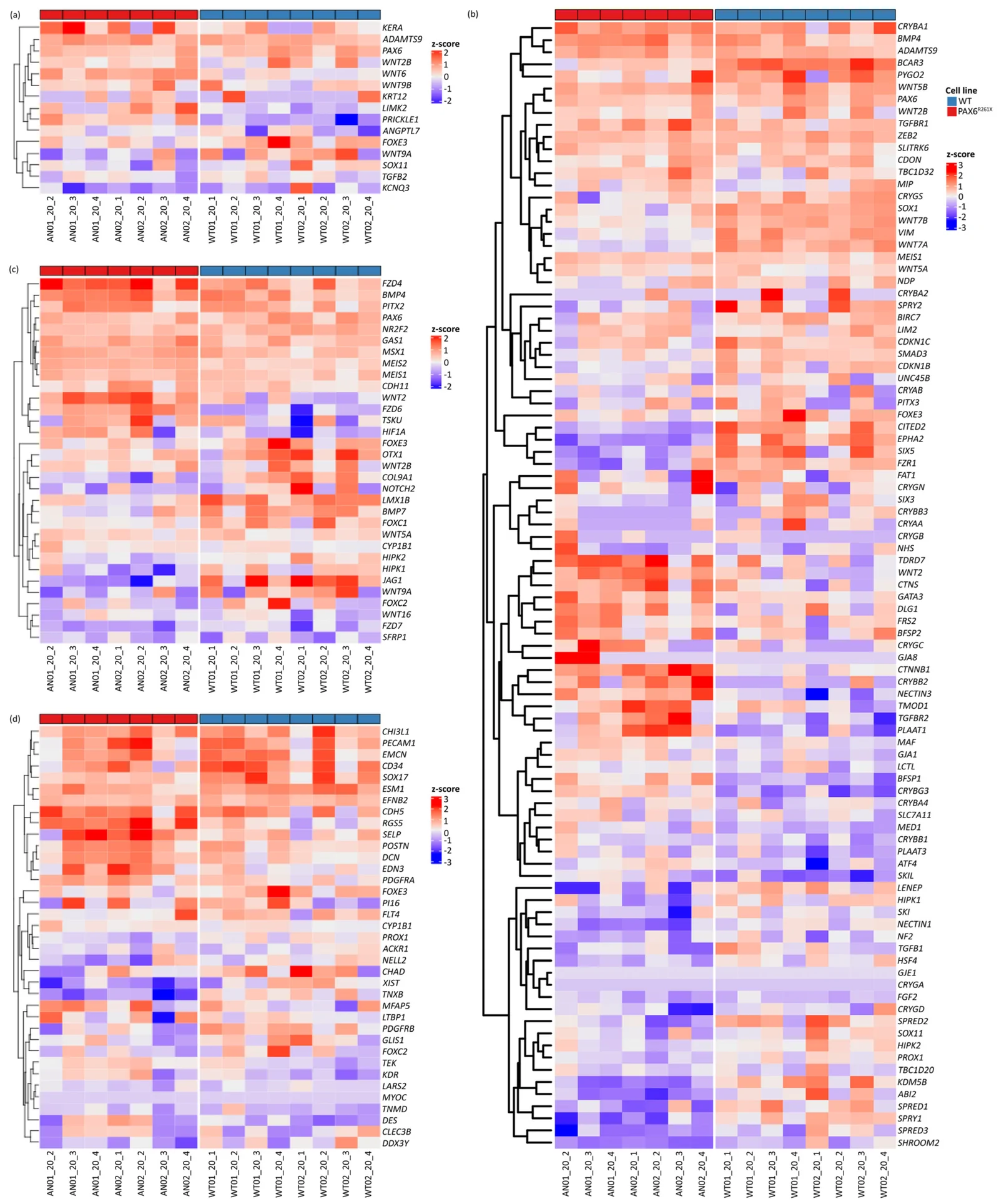
Heatmaps showing differential expression of anterior segment genes in PAX6^R261X^ optic vesicles compared with WT at day 20. (**a**) Corneal genes, (**b**) lens genes, (**c**) iris and ciliary body genes, (**d**) trabecular meshwork genes.

**Figure 4:**
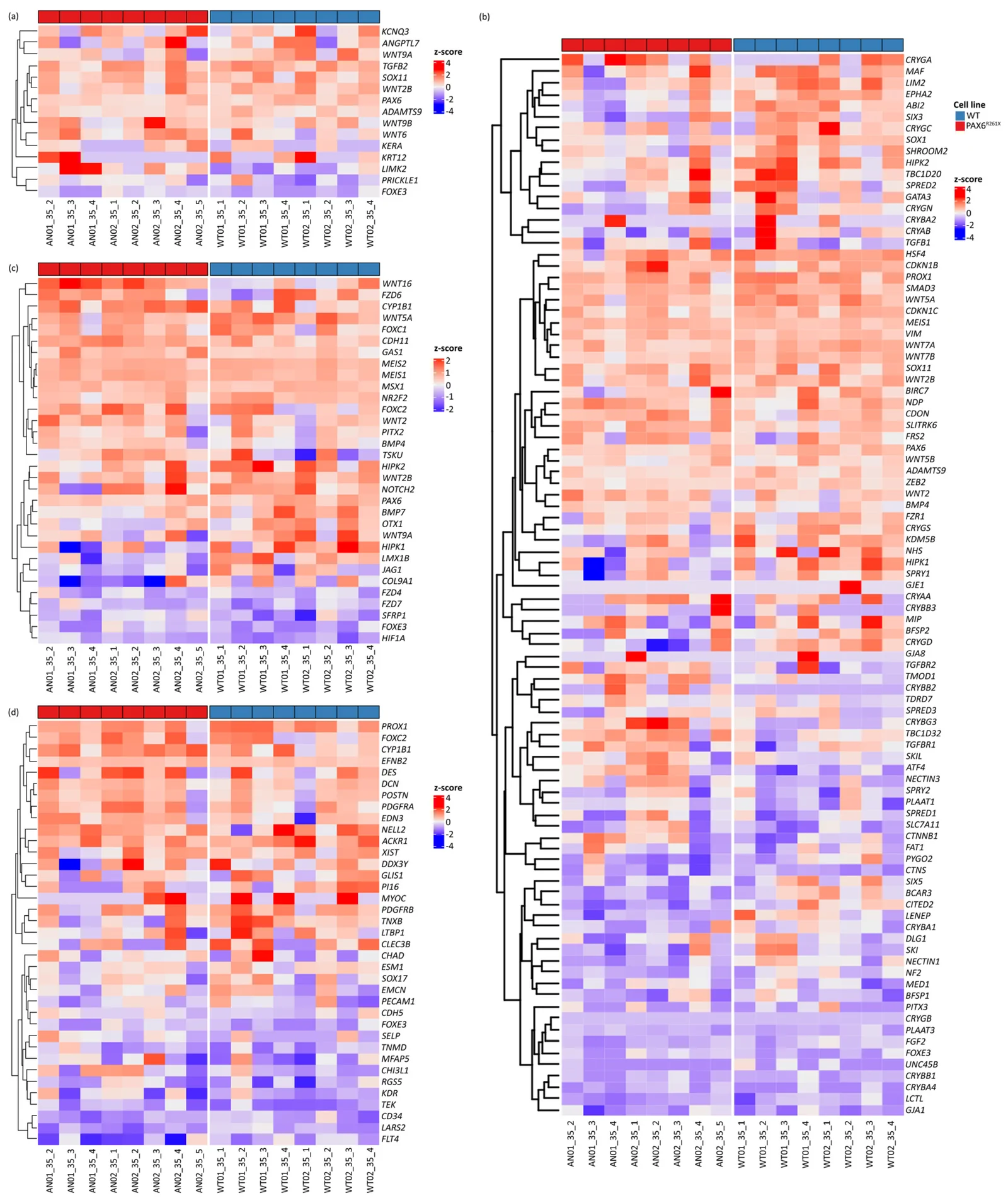
Heatmaps showing differential expression of anterior segment genes in PAX6^R261X^ optic vesicles compared with WT at day 35. (**a**) Corneal genes, (**b**) lens genes, (**c**) iris and ciliary body genes, (**d**) trabecular meshwork genes.

**Figure 5:**
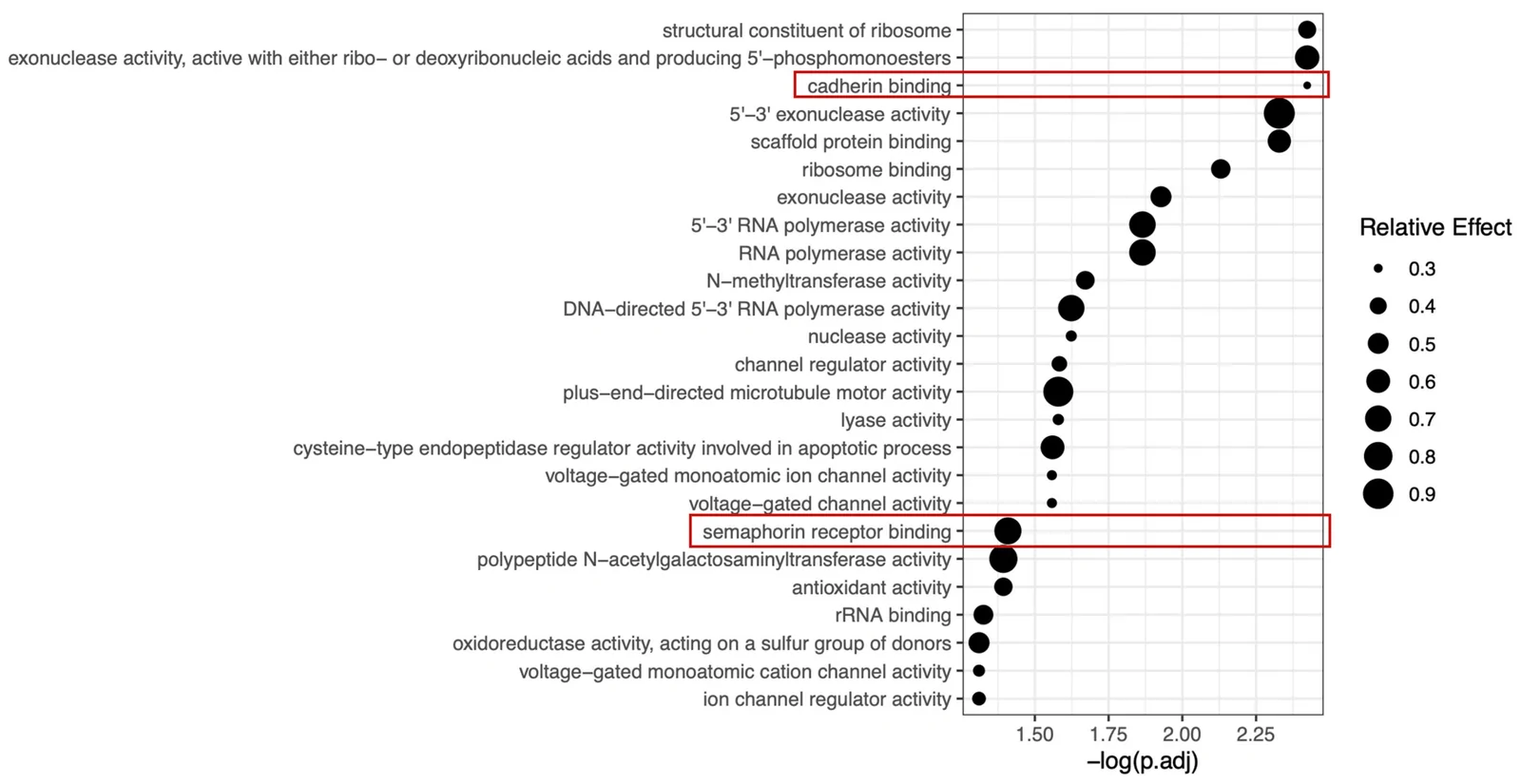
Gene ontology overrepresentation analysis: top 25 overrepresented pathways, with cadherin binding and semaphorin receptor binding pathways boxed. Relative effect: how many times larger is the overlap between the specified pathway and the list of differentially expressed genes than would have been observed by chance.

**Figure 6:**
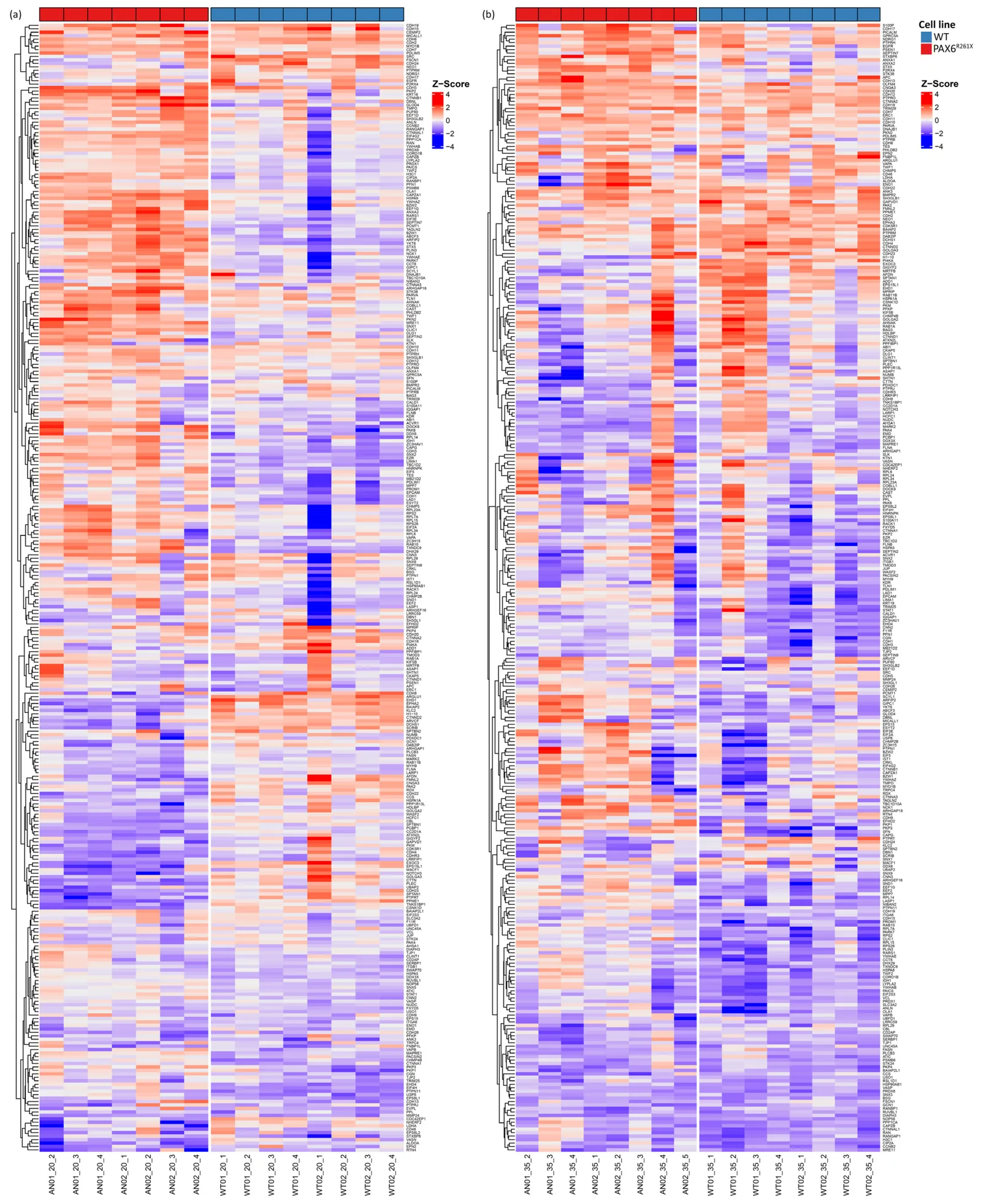
Heatmap showing differential expression of genes in the cadherin binding pathway in PAX6^R261X^ optic vesicles compared with WT. (**a**) Day 20, (**b**) day 35.

**Figure 7:**
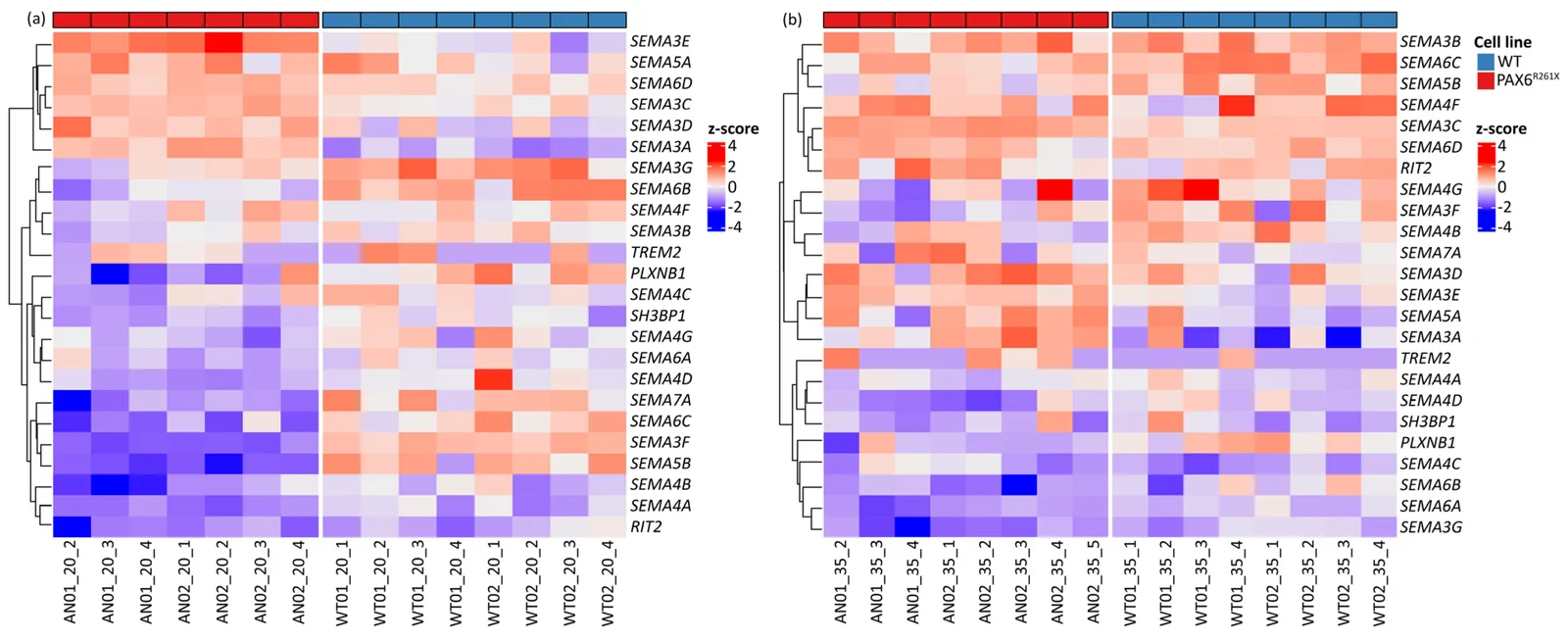
Heatmap showing differential expression of genes in the semaphorin receptor binding pathway in PAX6^R261X^ optic vesicles compared with WT. (**a**) Day 20, (**b**) day 35.

**Figure 8:**
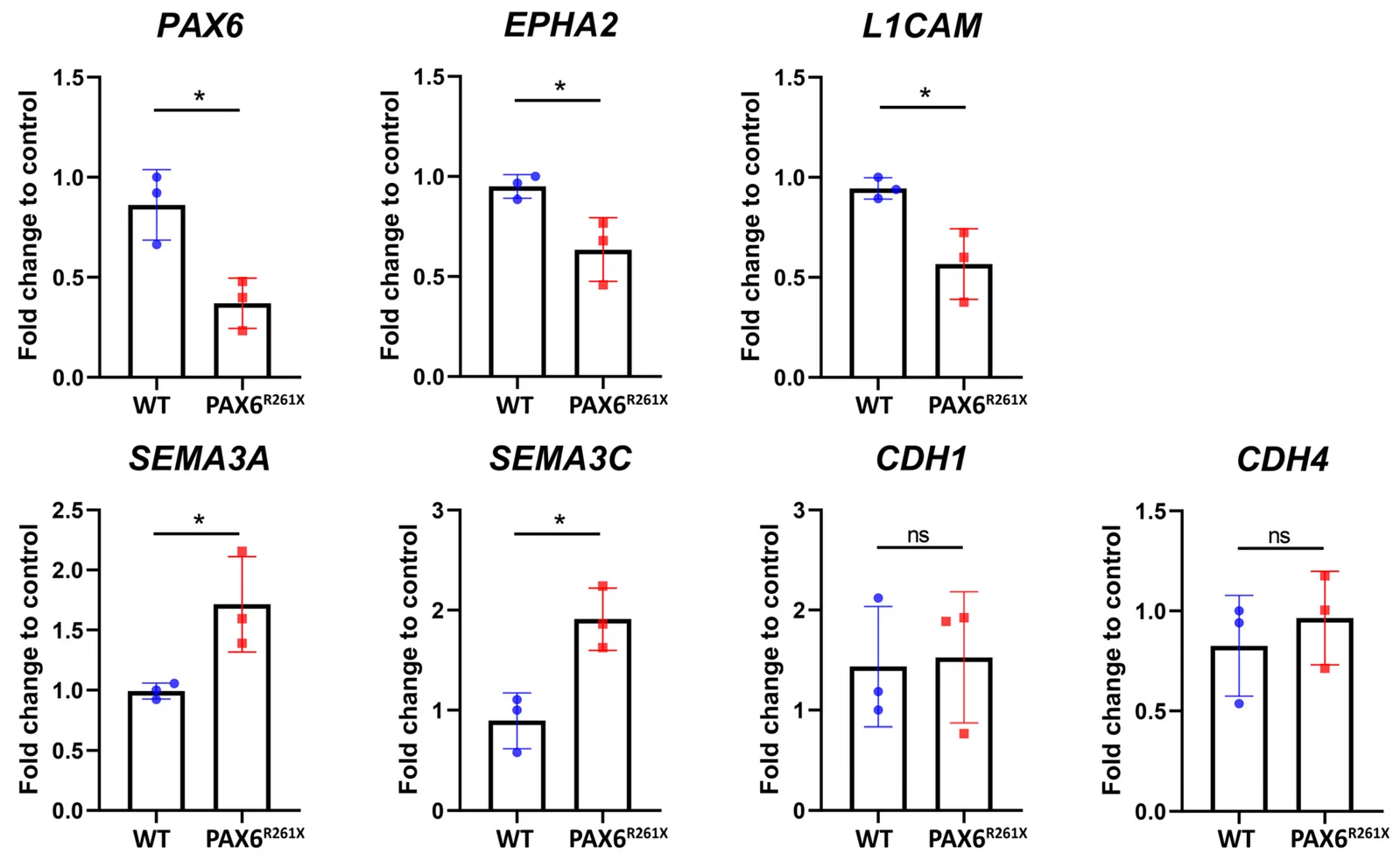
*EPHA2* and *L1CAM* are significantly downregulated while *SEMA3A* and *SEMA3C* are significantly upregulated in PAX6^R261X^ optic vesicles at day 35 compared to WT, confirming transcriptomic analysis. * *p* < 0.05; ns, not significant.

**Figure 9:**
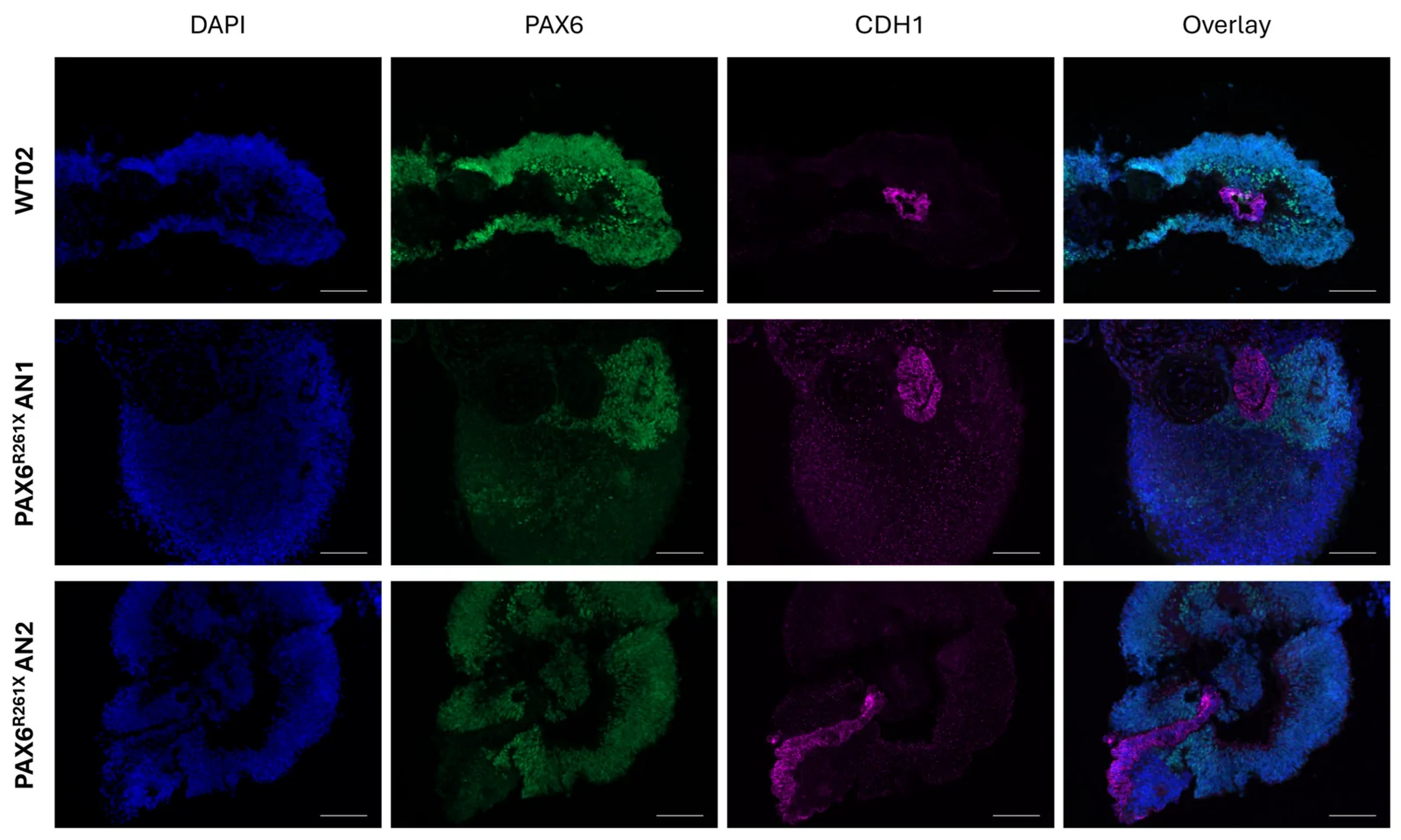
Immunofluorescence analysis of day 35 optic cups from WT and PAX6^R261X^ hiPSC, showing staining of DAPI (blue), PAX6 (green) and CDH1 (red). Scale bar, 100 μm.

**Table 1:** Primers used for RT-qPCR.

Gene	Forward Primer	Reverse Primer
*EPHA2*	GCATCAACCAGACAGAGCCC	GATGCTCCAGGAGACGCTAAG
*CDH1*	GGGTTAAGCACAACAGCAACAG	CTGACCCTTGTACGTGGTGG
*CDH4*	CAAACGGGACTGGGTCATCC	CTTTGTCGGACCGGATCCTC
*SEMA3A*	CGAGACCCTTACTGTGCTTG	GTCGTCTTGTGCGTCTCTTTG
*SEMA3C*	CCTGTTTCTCGGAACAGATCG	CTCCTCCAGAATGAGCTCGC
*L1CAM*	GCGGCAAATACTCAGTGAAGG	CTGTACTCGCCGAAGGTCTC

**Table 2:** Primary and secondary antibodies used for immunofluorescence staining.

Antibody	ID	Company	Dilution
*Primary antibodies*			
*CDH1*	610182	BD Bioscience	1:200
*EPHA2*	sc-398832	Santa Cruz	1:200
*SEMA3A*	Ab23391	Abcam	1:50
*CRX*	H00001406-M02	Abnova	1:100
*PAX6*	901301	Biolegend	1:300
*K18*	MAB3234	Millipore	1:400
*VSX2*	Ab16141	Abcam	1:100
*Secondary antibodies*			
Donkey anti-Mouse IgG Alexa Fluor 647	A-3157	Invitrogen	1:400
Goat anti-Rabbit IgG Alexa Fluor 488	A-11008	Invitrogen	1:400
Alexa Fluor 594 donkey anti-sheep IgG	A-11016	Invitrogen	1:400

## Data Availability

The original data presented in the study is available from NCBI Gene Expression Omnibus (GSE318345).

## Supplementary Materials

The following supporting information can be downloaded at: https://www.mdpi.com/article/10.3390/ijms27198449/s1.

## Abbreviations

The following abbreviations are used in this manuscript:

AAK	Aniridia-associated keratopathy
BRB	Blood–retinal barrier
EMT	Epithelial–mesenchymal transition
FAZ	Foveal avascular zone
hiPSC	Human-induced pluripotent stem cells
LFC	log_2_ fold change
NMD	Nonsense-mediated decay
padj	Adjusted *p*-value
RGC	Retinal ganglion cell
RPE	Retinal pigment epithelium
WAGR	Wilms tumour, aniridia, genital anomalies and a range of developmental issues
WT	Wild type
