# Supplementary material for: Early Eye Organoids Demonstrate Cadherin and Semaphorin Disruption in PAX6-Related Aniridia
Source: Int J Mol Sci. 2026 Sep 22;27(19):8449. doi: 10.3390/ijms27198449 (PMC13664914; doi:10.3390/ijms27198449)

Supplementary figures for "Early eye organoids demonstrate cadherin and semaphorin disruption in PAX6-related aniridia"

Supplementary Figure S1. Heatmap showing differentially expressed genes in aniridia hiPSC-derived organoids at each time point compared with WT day 0.

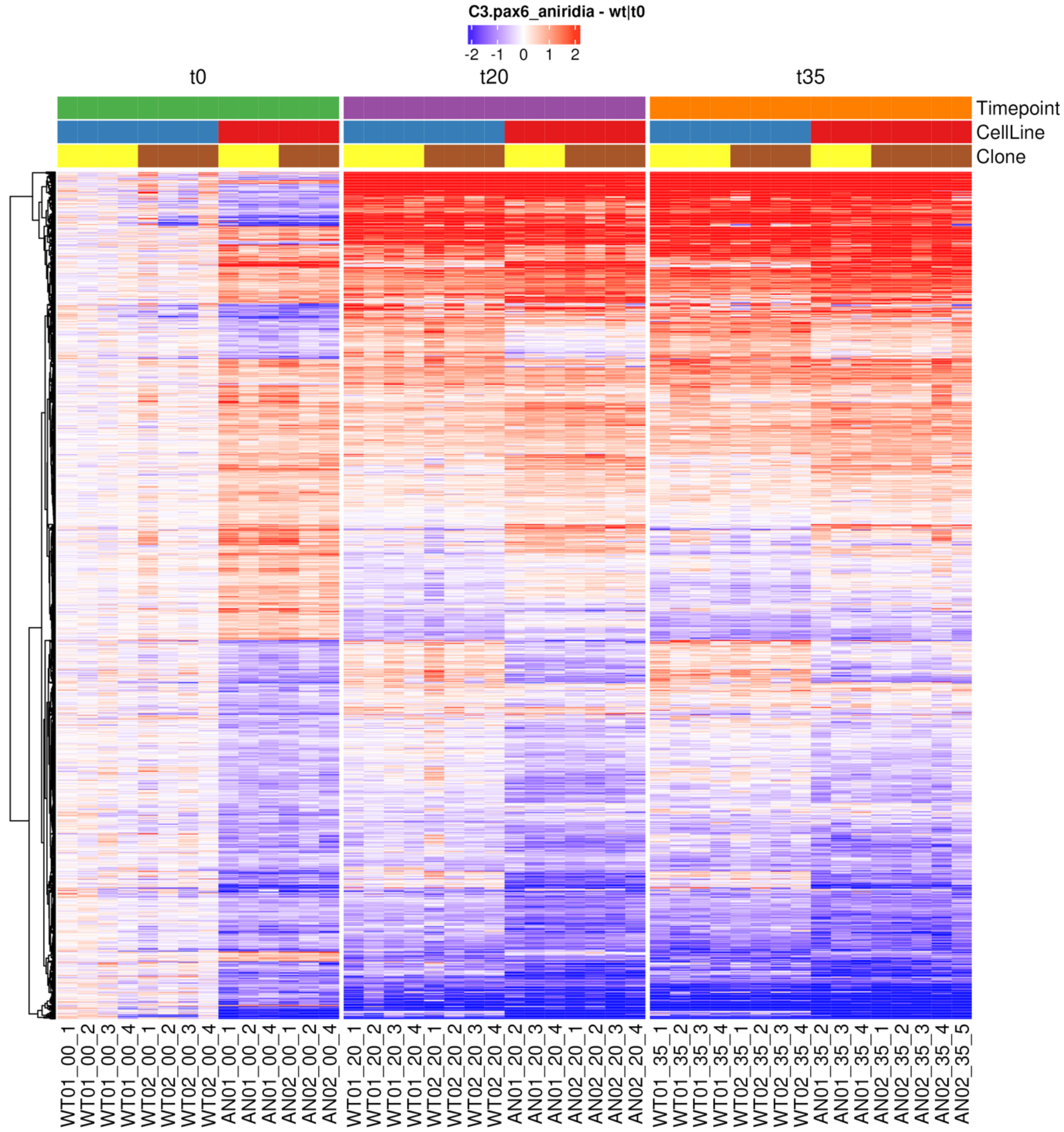

Supplementary Figure S2. PCA plot by clone and time point. Green area, day 0; purple area, day 20; yellow area, day 35.

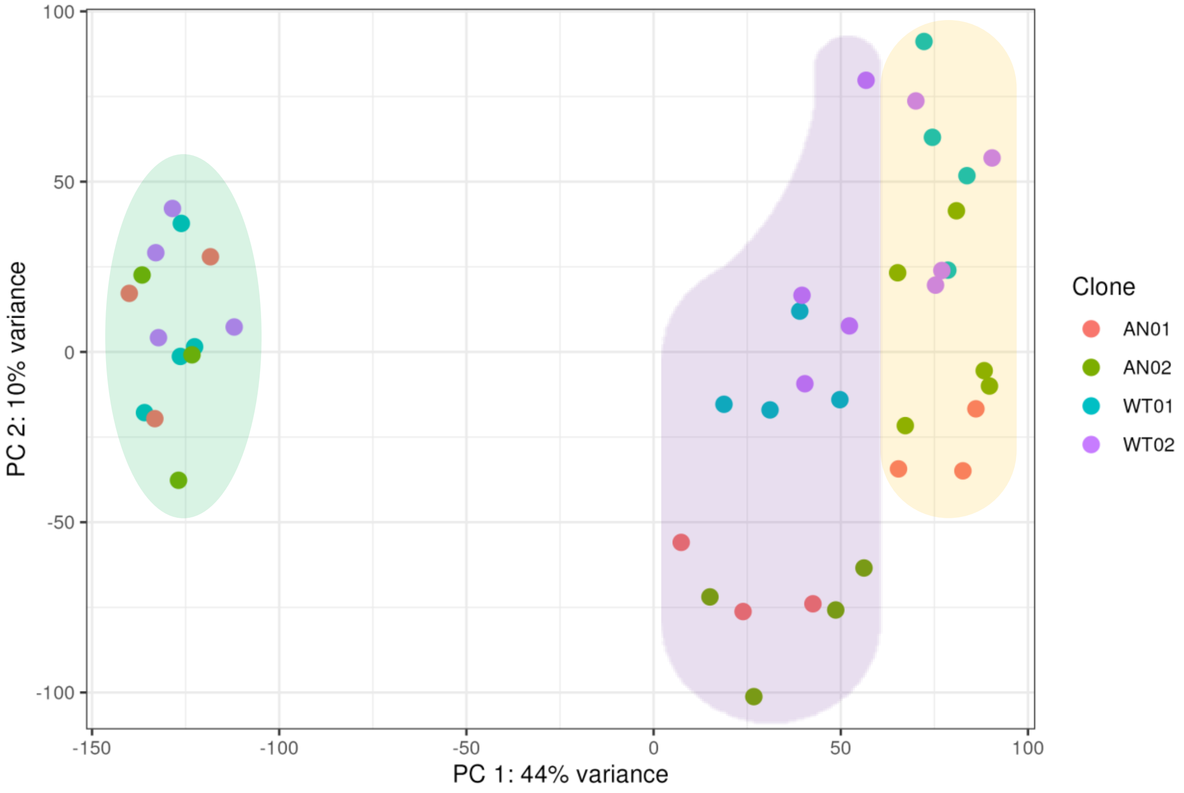

Supplementary Table S1. Anterior segment development pathways listed in Gene Ontology.

**Gene ontology code**	**Pathway name**
GO:0001743	lens placode formation
GO:0002088	lens development in camera-type eye
GO:0002089	lens morphogenesis in camera-type eye
GO:0002930	trabecular meshwork development
GO:0036331	avascular cornea development in camera-type eye
GO:0046619	lens placode formation involved in camera-type eye formation
GO:0060235	lens induction in camera-type eye
GO:0061072	iris morphogenesis
GO:0061073	ciliary body morphogenesis
GO:0061303	cornea development in camera-type eye
GO:0070306	lens fibre cell differentiation
GO:0070307	lens fibre cell development
GO:0070309	lens fibre cell morphogenesis
GO:1902746	regulation of lens fibre cell differentiation
GO:1902747	negative regulation of lens fibre cell differentiation
GO:1902748	positive regulation of lens fibre cell differentiation
GO:1903701	substantia propria of cornea development
GO:2001109	regulation of lens epithelial cell proliferation
GO:2001111	positive regulation of lens epithelial cell proliferation

Supplementary Table S2. Adjusted p values + log2FoldChange at day 20 and day 35 for major highlighted genes.

Gene symbol	Day 20		Day 35	
	padj	Log2FC	padj	Log2FC
CDH1	$9.33x10^{-5}$	1.92	0.0366	1.14
CDH4	$2.86x10^{-5}$	-1.69	0.0076	-1.16
L1CAM	$3.87x10^{-8}$	-1.89	0.0077	-1.02
SEMA3A	$3.06x10^{-3}$	1.21	0.0057	0.99
SEMA3C	$1.56x10^{-5}$	1.38	$1.35x10^{-3}$	1.27
SEMA3F	$1.52x10^{-11}$	-1.51	0.0083	-0.67

Supplementary Figure S3. Immunofluorescence analysis of day 35 optic cups from WT and $PAX6^{R261X}$ hiPSC, showing staining of DAPI (blue), PAX6 (green) and CDH1 (red). CDH1 was shown to co-localise with K18 positive cells (yellow). Scale bar, 100 μm.

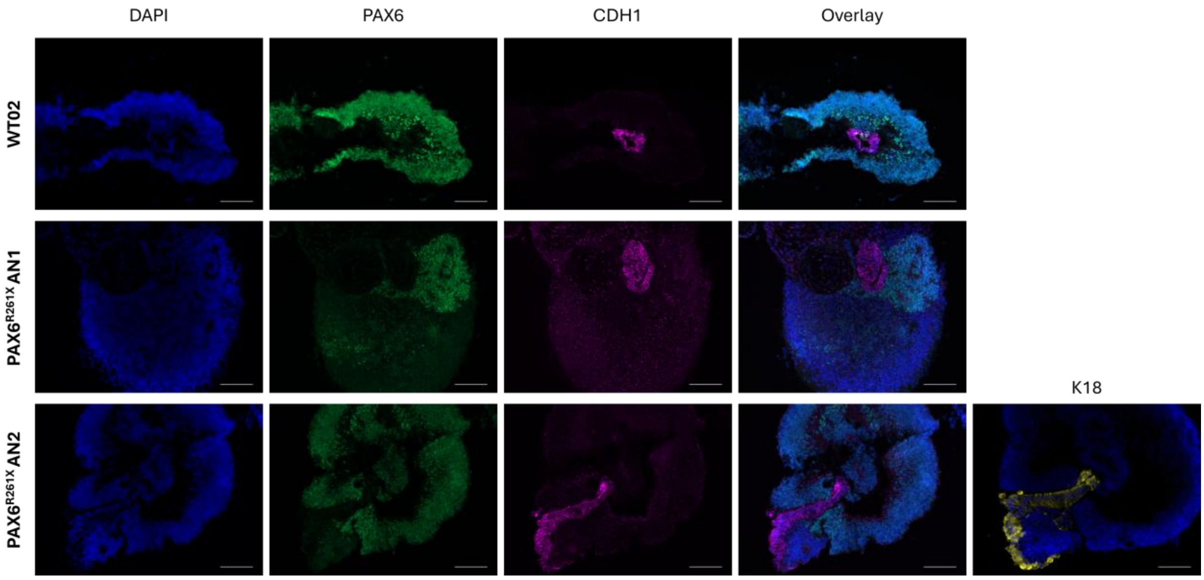

Supplementary Figure S4. Immunofluorescence staining of EPHA2 on day 35 optic cups from WT and PAX6^{R261X} hiPSC. DAPI (blue) was used for nuclei staining. Scale bar, 100 μm.

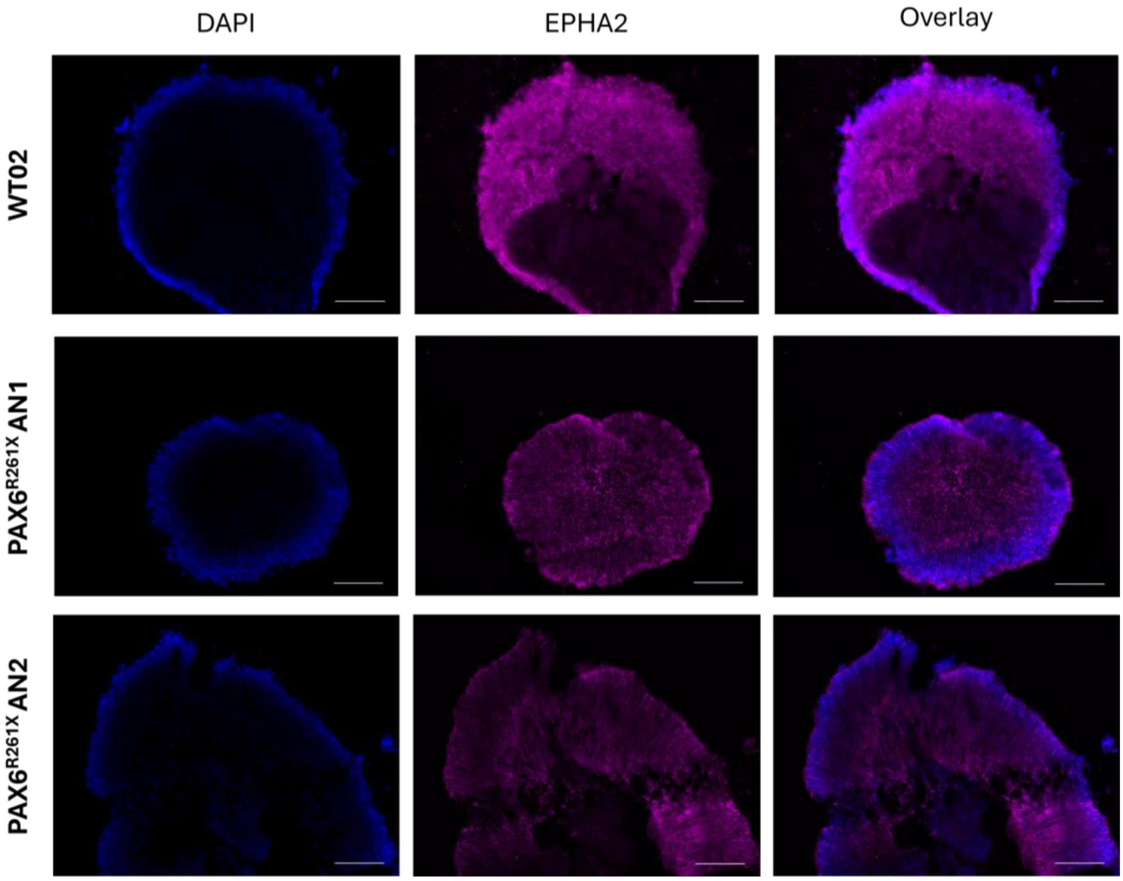

Supplement: Supplementary file 1 [file ijms-27-08449-s001.zip › ijms-3997381-supplementary.pdf]
